# Relative Validity and Reproducibility of a Food Frequency Questionnaire to Assess Nutrients and Food Groups of Relevance to the Gut Microbiota in Young Children

**DOI:** 10.3390/nu10111627

**Published:** 2018-11-02

**Authors:** Claudia Leong, Rachael W. Taylor, Jillian J. Haszard, Elizabeth A. Fleming, Gerald W. Tannock, Ewa A. Szymlek-Gay, Sonya L. Cameron, Renee Yu, Harriet Carter, Li Kee Chee, Lucy Kennedy, Robyn Moore, Anne-Louise M. Heath

**Affiliations:** 1 Department of Human Nutrition, University of Otago, P.O. Box 56, Dunedin 9054, New Zealand; leocl333@student.otago.ac.nz (C.L.); jill.haszard@otago.ac.nz (J.J.H.); liz.fleming@otago.ac.nz (E.A.F.); sonyalcameron@gmail.com (S.L.C.); yure1721@student.otago.ac.nz (R.Y.); carha847@student.otago.ac.nz (H.C.); cheli159@student.otago.ac.nz (L.K.C.); lucykennedy@hotmail.co.nz (L.K.); mooro526@student.otago.ac.nz (R.M.); 2 Department of Medicine, Dunedin School of Medicine, University of Otago, P.O. Box 56, Dunedin 9054, New Zealand; rachael.taylor@otago.ac.nz; 3 Nutrition Society of New Zealand, P.O. Box 2039, Whanganui 4543, New Zealand; 4 Department of Microbiology and Immunology, University of Otago, P.O. Box 56, Dunedin 9054, New Zealand; gerald.tannock@otago.ac.nz; 5 Microbiome Otago, University of Otago, P.O. Box 56, Dunedin 9054, New Zealand; 6 Institute for Physical Activity and Nutrition (IPAN), School of Exercise and Nutrition Sciences, Deakin University, Geelong, VIC 3220, Australia; ewa.szymlekgay@deakin.edu.au

**Keywords:** food frequency questionnaire, dietary fiber, microbiota, validity, reproducibility, children, New Zealand

## Abstract

Dietary fiber is an important nutrient for the gut microbiota, with different fiber fractions having different effects. The aim of this study was to determine the relative validity and reproducibility of a food frequency questionnaire (EAT5 FFQ) for measuring intake of fiber, and low and high fiber foods, in studies examining diet and gut microbiota in young children. One hundred parents of 5-year old children completed the 123-item EAT5 FFQ on two occasions four weeks apart. A 3-day weighed diet record (WDR) was completed on non-consecutive days between FFQ appointments. Mean correlations between the (randomly chosen) FFQ and WDR were acceptable for nutrient and food group intakes (*r* = 0.34 and *r* = 0.41 respectively). Gross misclassification was below chance (12.5%) for quartiles of nutrient (mean 5.7%) and food group (mean 5.1%) intake. ‘Absolute values for surrogate categories’ suggested the FFQ clearly differentiated between highest and lowest quartiles for all nutrients and food groups tested. Mean correlations between repeat administrations of the FFQ suggested very good reproducibility for nutrients (*r* = 0.83) and food groups (*r* = 0.80). The EAT5 FFQ appears to be an appropriate tool for investigating the intake of nutrients and food groups of relevance to the gut microbiota, and is the first FFQ validated to measure total, soluble and insoluble non-starch polysaccharide intakes in young children.

## 1. Introduction

A rapidly expanding literature suggests that the gut microbiota may have beneficial or harmful impacts on health [1,2]. Diet plays an important role in modulating gut microbiota, although much of this work has been in adults with little research undertaken in children [3,4]. A dietary component of particular interest is dietary fiber, as it is the main food source for the gut microbiota [5,6]. Because different classes (soluble, insoluble) or fractions (e.g., arabinoxylan from whole grains, pectin from fruits, and cellulose from vegetables) of fiber appear to impact gut microbiota in different ways [7,8,9,10], appropriate dietary assessment techniques must be used to improve understanding of how diet influences the microbiota and subsequent health outcomes. While weighed diet records (WDR) or 24-h recalls are generally considered gold standard methods of dietary assessment [11], they entail considerable respondent and researcher burden, and do not directly assess ‘usual’ intake. Food frequency questionnaires (FFQ) have lower respondent burden, estimate usual intake, and can be used in larger studies examining the long-term effects of diet on the gut microbiota. However, the validity of any new FFQ must be determined in order to ensure that it adequately measures the nutrients of interest in the relevant population [12].

In addition to investigating intake of nutrients, such as dietary fiber, it is also important to be able to determine intake of foods. Most foods are complex combinations of multiple nutrients and food components that cannot be captured by simply measuring nutrient intake, we eat foods rather than nutrients, and dietary guidelines refer to foods rather than nutrients [12].

To date, no studies have validated an FFQ specifically designed to look at both nutrients and food groups of relevance to the gut microbiota, specifically dietary fiber [9], soluble and insoluble non-starch polysaccharides (NSP) [7,8], and food groups that are high and low in fiber. Therefore, the aim of this study was to determine the relative validity and reproducibility of the EAT5 FFQ for estimating intake of both nutrients and food groups of relevance to the gut microbiota in 5-year old New Zealand children.

## 2. Materials and Methods

### 2.1. Study Design

The study was designed to validate the EAT5 FFQ for measuring intake of nutrients (energy, carbohydrate, fiber, total NSP, soluble NSP, and insoluble NSP) and food groups (e.g., ‘Higher fiber more healthy cereals’, ‘Higher fiber less healthy cereals’, ‘Lower fiber more healthy cereals’, ‘Lower fiber less healthy cereals’, ‘Nuts and legumes’, ‘Fruits’, ’Vegetables’, ‘Potatoes and hot chips’, ‘Yoghurt’). Parent and child participants attended two appointments four weeks apart. At the first appointment, the EAT5 FFQ and socio-demographic questionnaire were completed by the parents, and anthropometric measurements of the child were obtained. A 3-day WDR was completed over the following four weeks. At the second appointment, the EAT5 FFQ was administered again so that reproducibility could be assessed. The FFQs asked about intake in the past month.

### 2.2. Participants

A convenience sample of 100 participants (parent-child pairs) was recruited from Dunedin, Auckland and Wellington (New Zealand) from February 2015 to December 2017. The child had to be healthy and aged ≥5 to ≤6 years during the time of assessment to be eligible for the study. The study was conducted in accordance with the Declaration of Helsinki. The Human Ethics Committee of the University of Otago, Dunedin, New Zealand, granted ethical approval for the study (reference number H14/154). Written informed consent was obtained from all parents and children.

Parents completed a questionnaire on their child’s age, sex, ethnicity and number of siblings. Using the participants’ home address, the NZDep2013 Index of Deprivation was determined (range from 1 to 10, with a value of 1 representing the least deprived 10% of New Zealand households, and a value of 10 representing the 10% most deprived) [13]. The child’s height and weight were measured using standard protocols [14]. Height was measured using a Leicester wall stadiometer (Tanita, IL, USA) to the nearest 0.1 cm, with duplicate measures taken (and a third measurement if duplicates were not within 0.7 cm of each other). Weight was measured using digital scales (Seca Alpha model 770; Seca, Hamburg, Germany) to the nearest 0.1 kg, with duplicate measures taken (and a third measurement if duplicates were not within 0.1 kg of each other). Body mass index (BMI) was calculated from the average of height and weight measurements using the formula: weight in kilograms divided by height in meters squared. 

### 2.3. EAT5 Food Frequency Questionnaire

The EAT5 FFQ was designed to be quantitative, interviewer administered and to rank 5-year old children by intake over the past month of nutrients of relevance to the gut microbiota, with data collected from the primary caregiver (usually a parent). The EAT5 FFQ was a modified version of the previously validated EAT FFQ designed to assess nutrient intake [15] and dietary patterns [16] in New Zealand toddlers aged 12–24 months, which was in turn originally based on the Southampton Women’s Survey questionnaire for infants [17]. For the EAT5 FFQ, the EAT food list was reconstructed to remove infant foods and to include a wider variety of fruit and vegetable food items in order to better differentiate between fiber fractions (e.g., soluble vs. insoluble NSPs). Three important components were present in the EAT5 FFQ: (i) cross-check questions for fruit and vegetable intake; (ii) use of volume for the amount eaten for foods that did not have a natural portion size (e.g., ‘slice’) with participants demonstrating volumes consumed using dried beans and rice on plates, bowls and cups; and (iii) a wide variety of fruit and vegetable food items—14 and 18 food items respectively. The cross-check question asked parent participants the overall frequency of their child’s fruit (or vegetable) consumption over the past month, so that the frequency of each individual item within the fruit (or vegetable) section could be weighted to adjust for the overall frequency (i.e., ‘fruit and vegetable adjusted’ frequency) [18]. The EAT5 FFQ fruit (or vegetable) weighting factor was calculated as follows: ‘frequency from cross-check question’ divided by ‘sum of frequencies from the fruit (or vegetable)’. The weighting factor was then applied to the frequency for each fruit (or vegetable) item to get the ‘fruit and vegetable adjusted’ frequency value for that individual fruit (or vegetable) item. This was undertaken to account for the well-established overestimation in fruit and vegetable intake that can occur when participants estimate and report consumption frequency for multiple individual fruits and vegetables [19,20]. 

The EAT5 FFQ asked about intakes over the past month using 10 frequency-response options, ranging from ‘not eaten this month’ to an open-ended question for multiple times per day. The EAT5 FFQ comprised 123 food items under 11 section headings: (i) bread, crackers and breakfast cereals; (ii) rice and pasta; (iii) fruits; (iv) vegetables; (v) meat, chicken, fish, eggs, beans; (vi) spreads; (vii) cakes, biscuits, snacks; (viii) milk and dairy products; (ix) puddings; (x) drinks; (xi) takeaways. The EAT5 FFQ is available upon request from the corresponding author.

Nutrient intakes were calculated using FOODfiles 2014 [21], except for NSP values where FOODfiles 2010 [22] was used because these data were not available for all foods in FOODfiles 2014. Some of the 123 food items had multiple foods in the same food item question. For these food items, the nutrient composition of the individual foods was weighted using age-appropriate frequency and portion size consumption data [23,24].

### 2.4. Weighed Diet Record

Parents completed a 3-day WDR on 3 randomly assigned, non-consecutive days (1 weekend day and 2 week days) over four weeks. Participants were given detailed verbal and written instructions and a calibrated electronic kitchen scale (Salter Vista, Kent, UK; ±1 g) at the first visit and then contacted during the collection period so that they could ask further questions. On the second visit, the WDR was collected and checked by trained staff. Diet records were analyzed with the Kai-culator nutritional software package version 1.16a (Department of Human Nutrition, University of Otago, New Zealand) using the nutrient database FOODfiles 2014 [21], except for NSP values where FOODfiles 2010 [22] was used.

### 2.5. Food Groups

**Figure 1:**
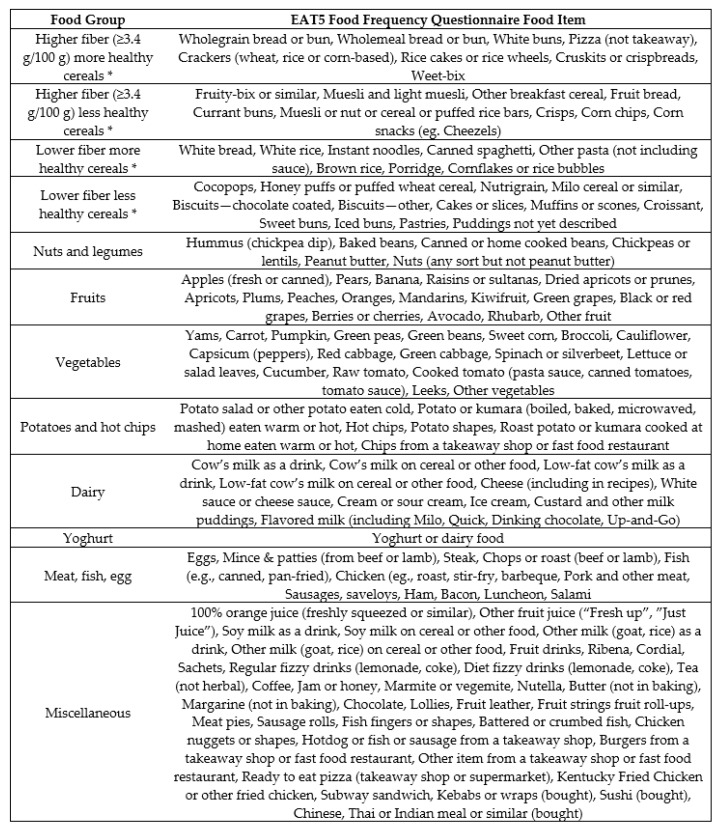
Food groups developed based on their relevance to the gut microbiota. Note: * ‘Higher fiber’ was defined as ≥3.4 g of dietary fiber which was the median fiber content of the 1010 individual food items reported in the weighed diet records ; ‘More healthy’ was defined as ‘staple foods’ with a lower saturated fat and sugar (<15 g/100 g) content.

The 123food items in the EAT5 FFQ were assigned to 12 food groups that were defined based on food groups of relevance to the gut microbiota [4,7,25,26], and the number of consumers (i.e., at least 8 consumers were required in each food group so that there would be sufficient power to perform the food group analyses [27]). Food items were allocated to the food groups based on nutrient profile and similarity of use (Figure 1). The same 12 food groups were used for the 3-day WDR data. In total, 1010 individual food items were entered into Kai-culator from the WDRs. These 1010 individual food items were allocated to the 12 food groups, except for water, which was excluded.

### 2.6. Statistical Analysis

Data were analyzed using Stata statistical software (version 13; StataCorp, College Station, TX, USA). A *p*-value of *p* < 0.05 was considered to indicate statistical significance.

The EAT5 FFQ used for the validation analysis was randomly chosen from the first or second FFQ administered. Data reported in the main text uses the ‘fruit and vegetable adjusted’ EAT5 FFQ values, which were calculated using a weighting factor to adjust the individual fruit and vegetable food items for the participant’s overall consumption of fruits and vegetables (see above). Crude EAT5 FFQ values can be found in the supplementary material (Tables S1–S6). Histograms were plotted for each variable and used to visually assess the normality of their distribution. The majority of the distributions were right-skewed so geometric means and 95% confidence intervals (CI) were used. However, the majority of the distributions of the paired differences were normally distributed. Spearman’s correlation coefficients were calculated comparing the FFQ with the WDR. Correlations of 0.30–0.49 were considered ‘acceptable’, 0.50–0.70 ‘good’ [12], and >0.70 were considered ‘very good’. The WDR data were adjusted for intra-individual variation using the Multiple Source Method (MSM) program [28] in order to provide a better estimate of ‘usual intake’. Cross-classification of WDR and FFQ quartiles was also carried out. The percentage of participants correctly classified was defined as the FFQ categorizing the diet into the same quartile as the WDR, while gross misclassification was defined as the FFQ categorizing the diet into the highest quartile when the WDR was categorized into the lowest quartile, and vice versa. The ‘absolute values for surrogate categories’ approach determines the extent to which intakes measured using a new method (EAT5 FFQ) reflect intakes measured using a reference method (WDR). Actual values for the surrogate categories [12] were calculated as follows: participants were assigned to quartiles according to intake estimated by the EAT5 FFQ, then the mean intake in each quartile was calculated using the intake determined by the WDR. Regression analyses were calculated to see if there was a trend in the step-wise increases across the quartiles, and the difference in quartile 1 to quartile 4. Bland-Altman analyses [29] were used to assess the agreement between the FFQ and WDR at the individual level.

Intra-class correlation coefficients were calculated comparing the first and second administration of the EAT5 FFQ to assess reproducibility, with correlations of 0.30–0.49 considered ‘acceptable’, 0.50-0.70 ‘good’ [12], and >0.70 ‘very good’.

## 3. Results

### 3.1. Study Population

One hundred participants were recruited, of whom 99 parent-child pairs completed the two FFQs and the 3-day WDR. One parent-child pair completed only the first FFQ and the 3-day WDR, meaning that 100 participants were included in the validity analysis and 99 participants in the reproducibility analysis. The 100 young children (44% male) had a mean (range) age of 5.5 (4.9–6.0) years and BMI of 16.0 (13.7–19.7) kg/m^2^. The participants were mainly of New Zealand European ethnicity (80%), with 13% Māori and 5% Asian. Half of the participants (49%) had one sibling, and 31% more than one. According to the NZDep2013 Index of Deprivation [13], 19% of the participants were from households in the three most deprived deciles (compared to the expected 30%). 

### 3.2. Relative Validity and Reproducibility of Nutrient Intakes

In general, the ‘fruit and vegetable adjusted’ FFQ data were closer to the WDR data than the crude unadjusted FFQ data so are used to describe the performance of the EAT5 FFQ. The crude unadjusted figures are presented in the supplementary material (Tables S1–S6).

Table 1 shows the energy and selected nutrient estimates from the EAT5 FFQ compared to the WDR. There were no significant differences in mean carbohydrate, fiber and total NSP intakes measured by the EAT5 FFQ and the WDR. Data comparing the crude EAT5 FFQ and WDR data can be found in the supplementary material (Table S1). Estimates of intakes of macro- and micronutrients of less relevance to the gut microbiota can be found in Table S2.

The mean correlation between nutrients measured by the EAT5 FFQ and WDR was 0.34 (‘acceptable’), with a range from 0.24 for soluble NSP to 0.38 for total and insoluble NSP (Table 2). The correlations were slightly higher for MSM adjusted values with a mean of 0.35 (‘acceptable’). The correlations for the crude EAT5 FFQ and other nutrients can be found in Tables S3 and S4. Table 2 also reports the correlations used to assess reproducibility of the first and second administration of the EAT5 FFQ. The mean correlation was 0.83 (‘very good’), with a range from 0.80 to 0.88. The reproducibility correlations for the crude EAT5 FFQ and other nutrients can be found in Tables S3 and S4.

All nutrients and energy had a percentage correctly classified into quartiles by the EAT5 FFQ and WDR that was greater than chance (25%), ranging from 28% (fiber and insoluble NSP) to 36% (carbohydrate) (Table 3). The mean percentage grossly misclassified was 5.7% and correctly classified to extreme quartiles was 19.1% (12.5% would be expected by chance alone). The percentage cross-classifications for the crude EAT5 FFQ and other nutrients can be found in Tables S5 and S6.

Trends for the actual values for surrogate categories show the expected increase across the FFQ quartiles for energy and nutrients (all *p* ≤ 0.005) (Table 4). The EAT5 FFQ clearly differentiated between the first and fourth quartile for energy and nutrients (all differences between the first and fourth quartile *p* ≤ 0.011).

However, the limits of agreement between the FFQ and the WDR were wide for energy and all five nutrients (Table 1).

**Figure 2:**
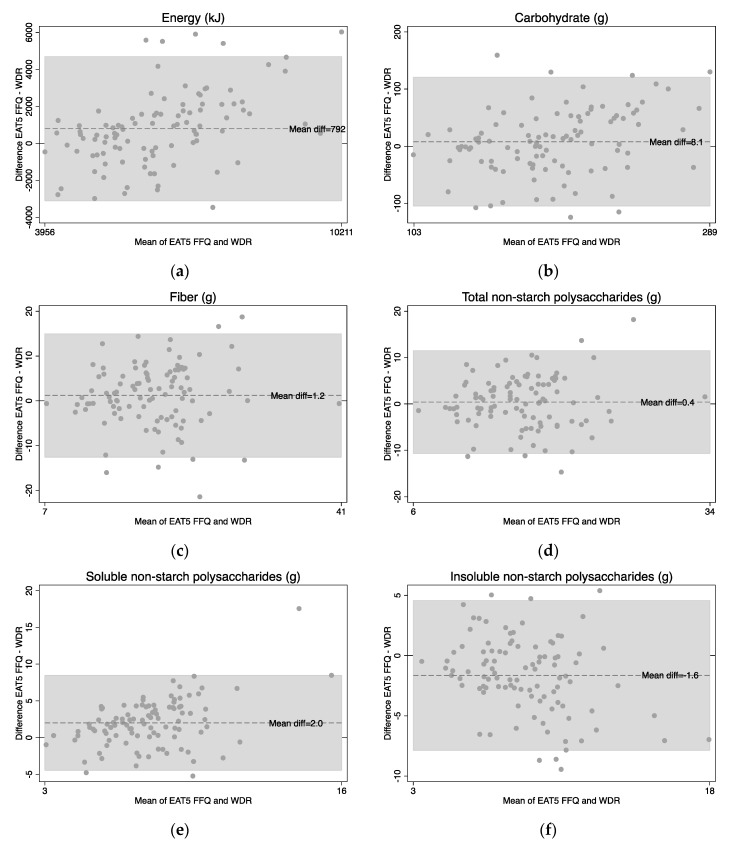
Bland-Altman plots for nutrient intakes from a randomly chosen FFQ1 or FFQ2 EAT5 FFQ (‘fruit and vegetable adjusted’) and the weighed diet record (WDR): (**a**) Energy in kJ; (**b**) Carbohydrate in grams; (**c**) Fiber in grams; (**d**) Total non-starch polysaccharides in grams; (**e**) Soluble non-starch polysaccharides in grams; and (**f**) Insoluble non-starch polysaccharides in grams.

Bland-Altman plots show little bias in the EAT5 FFQ with a good scatter seen in the plots of energy and nutrients (Figure 2), although possible overestimation of energy and soluble NSP at higher intakes was indicated.

### 3.3. Relative Validity and Reproducibility of Food Group Intakes

Table 5 shows the mean energy contribution from each food group for the EAT5 FFQ compared to the WDR. For six of the 12 food groups, the mean EAT5 FFQ intake was not significantly different from the WDR. The other six foods groups gave significantly higher estimates than the WDR. Estimates of intakes of mean amount eaten in grams from each food group can be found in Table S7.

The mean correlation for food group intakes between the EAT5 FFQ and WDR was 0.41 (‘acceptable’), with a range from 0.28 for ‘vegetables’ and ‘miscellaneous’ to 0.56 (‘good’) for ‘meat, fish, egg’ (Table 6). Table 6 also reports the correlations used to assess reproducibility of the estimates of food group intake for the first and second administration of the EAT5 FFQ. The mean correlation was 0.80 (‘very good’), with a range from 0.57 (‘potatoes’: ‘good’) to 0.91 (‘low fiber more healthy cereals’: ‘very good’). The relative validity and reproducibility correlations for the amount eaten in grams from each food group can be found in Table S8.

All food groups had a percentage correctly classified into quartiles by the EAT5 FFQ and WDR that was greater than chance (25%), with a range from 28% (‘vegetables’) to 51% (‘meat, fish, egg’) (Table 7). The mean percentage grossly misclassified was 5.1% and correctly classified to extreme quartiles was 22.8% (12.5% would be expected by chance alone). The percentage cross-classifications for the amount eaten in grams from each food group can be found in Table S9.

Trends for the actual values for surrogate categories show the expected increase across the FFQ quartiles for all food groups (all *p* ≤ 0.028) (Table 8). The EAT5 FFQ clearly differentiated between the first and fourth quartile for all food groups (all differences between the first and fourth quartile *p* ≤ 0.033).

However, the limits of agreement were wide for all food group intakes (Table 5).

Bland-Altman plots show some bias in the food group estimates from the EAT5 FFQ with the FFQ having less agreement with the WDR at higher intakes (Figure S1).

## 4. Discussion

The EAT5 FFQ was designed to measure intakes of nutrients and food groups of relevance to the gut microbiota in 5-year old children and showed acceptable validity, and very high reproducibility, for these over a 4-week period. The FFQ provided good estimates of mean intakes of carbohydrate, fiber and total NSP intake, although it tended to overestimate energy (by 14%) and soluble NSP (32%) and underestimate insoluble NSP (by 18%) compared to the WDR; ranked most intakes acceptably (measured by correlation); and was able to differentiate well between categories of intake. Specifically, the EAT5 FFQ assigned children to correct quartiles of intake well, with very few children being grossly misclassified into the opposite quartile of intake, and was able to clearly differentiate between low and high intakes identified in the WDR. 

It is difficult to compare our results directly with the literature given that no FFQs have been validated to specifically measure nutrients and foods of relevance to the gut microbiota in children. However, several validation studies have measured intake of energy and nutrients such as carbohydrate and fiber. The correlations we observed in the current study for these nutrients were within the range of those obtained in previous FFQ validation studies in young children [33,38,39,40,41,42]. Adjusting for usual intake (using MSM) resulted in a small improvement in correlation values. The cross-classification results for energy, carbohydrate and fiber were similar to [31] and better than [39] other FFQ validation studies in young children that have reported these data. Unfortunately, the food group correlations and cross-classifications cannot be compared to previous studies because food groupings depend on the nutrients of focus for the specific validation study, so are different for different studies. However, we believe the FFQ performs well in this context, with correlation values and gross-misclassification values for the food groups being comparable to those obtained for the nutrients in this study.

‘Absolute values for surrogate categories’ showed the expected stepwise increase for all nutrients and food groups and suggested that the EAT5 FFQ clearly differentiated between highest and lowest quartiles for all nutrients and food groups tested. The ‘absolute values for surrogate categories’ approach was developed by Willett [12], and although it has not been commonly reported, it has been used in the validation of calcium intakes in children [43] and iron intakes in adults [44]. It is a useful validation tool as it indicates the extent to which an FFQ is able to differentiate between broad categories of intake, as is often required in epidemiological studies.

The reproducibility of the EAT5 FFQ was consistently high, with mean correlations for reproducibility for nutrients of 0.83 and food groups of 0.80. This was higher than the range of 0.26 to 0.78 that was found for the same nutrients in other FFQ validation studies [31,39,41]. Bland-Altman plots for nutrients showed little bias in the EAT5 FFQ with a good scatter seen in the plots, but there was some bias for the food groups, particularly at higher intakes. As expected of an FFQ, the EAT5 FFQ had wide limits of agreement for nutrients and food groups suggesting that it is not appropriate for determining nutrient or food intake in individuals.

This study has several strengths. First, the EAT5 FFQ is the first FFQ validated to measure total, soluble and insoluble NSP intakes, and food groups of relevance to the gut microbiota, in children. The only other studies that have validated FFQs for nutrients of relevance to the gut microbiota (i.e., intake of NSPs [45], dietary fiber [46,47], inulin and oligosaccharides [48]) have been in adults. Interestingly, our study had lower correlations (i.e., they were ‘acceptable’) than the correlations that were obtained in an adult study, validating intake of NSPs (which were ‘acceptable’ to ‘good’) [45]. A possible explanation may be that parental proxy reporting acts as an additional layer of potential error in assessing diets in children. However, the studies validating dietary fiber intake in adults [46,47] also used another questionnaire as the reference method, rather than a diet record. This means that they did not use a widely accepted method of dietary assessment with different errors to those of the FFQ as a reference method, as is recommended [49], and as was used in the current study. Second, we aimed to validate several carbohydrate food groups of interest defined by their dietary fiber content and overall healthiness using strict criteria. By contrast, most previous studies have combined all carbohydrate containing foods into a single group such as cereal or grains [16,50] with most of the ‘less healthy’ carbohydrate foods appearing in the ‘snacks’ food group, even if they contained fiber [51]. Third, we used a non-consecutive 3-day WDR as the reference method. Many other FFQ validation studies in children used 24-h recalls [31,33,38,39,41,42] or estimated diet records [32], both of which have similar errors to an FFQ with potential for memory lapses and errors in portion size estimation. Finally, cross-check questions were used for the fruit and vegetable sections. Using the cross-check questions improved the performance of the EAT5 FFQ (unadjusted values can be found in the supplementary material). This is particularly important, as the EAT5 FFQ was developed to look at nutrients of relevance to the gut microbiota and hence has a large number of fruit and vegetable questions. Fruit and vegetable food groups have been shown to be commonly over-reported in other FFQ validation studies [18].

Our study has some limitations. First, the ethnicity of the participants is not representative of the New Zealand population as a whole, with a higher proportion of New Zealand Europeans, and an over-representation of participants from the lower and middle deciles of household deprivation. Second, the FFQ was administered only to the primary caregiver, and as the children were five years of age, they would be attending school, so were not with their parents at all times. However, in an effort to overcome this common limitation, parents were asked to report any food and amount eaten if someone else provided their child with food, and the child attended the appointment with their parent so was available for parents to clarify their answers. Third, it was only possible to test the ability of the FFQ to measure intake of fiber and total, soluble and insoluble NSPs, not intake of smaller fiber fractions such as arabinoxylan and pectin that may also impact on the gut microbiota. This was because these fractions are not measured and reported in the New Zealand food composition database, FOODfiles, and the literature was not sufficient to provide reliable data for all 1010 foods consumed in the WDRs.

## 5. Conclusions

In conclusion, the EAT5 FFQ has acceptable validity when compared with a 3-day WDR and has very good reproducibility when measured over four weeks. It is suitable for assessing mean absolute intake of carbohydrate, fiber, and total NSP. The EAT5 FFQ is able to rank the diets of young children adequately, and to correctly assign low and high intakes of nutrients and food groups of relevance to the gut microbiota. The EAT5 FFQ is therefore an appropriate dietary assessment tool for investigating intake of nutrients and food groups of relevance to the gut microbiota in studies of young children.

## Figures and Tables

**Table 1:** Mean daily intake, mean difference, and limits of agreement for selected nutrients of relevance to the gut microbiota according to WDR and EAT5 FFQ in 5-year old children (*n* = 100) ^1,2^.

Nutrient	WDR		EAT5 FFQ		EAT5 FFQ vs. WDR			
	Mean ^3^	(95% CI)	Mean ^3^	(95% CI)	Mean Diff	(95% CI)	*p* ^4^	LOA ^5^
Energy (kJ)	5845	(5613, 6086)	6476	(6107, 6866)	792	(398, 1187)	<0.001	−3183–4768
Carbohydrate (g)	181	(173, 190)	186	(175, 197)	8.1	(−3.3, 19.4)	**0.163**	−107–123
Fiber (g)	18	(17, 19)	19	(18, 20)	1.2	(−0.2, 2.6)	**0.090**	−13–15
Total NSP (g)	15	(14, 16)	16	(15, 17)	0.4	(−0.7, 1.5)	**0.488**	−11–12
Soluble NSP (g)	6.3	(5.9, 6.6)	8.0	(7.4, 8.6)	2.0	(1.4, 2.7)	<0.001	−4.6–8.6
Insoluble NSP (g)	8.9	(8.3, 9.6)	7.5	(7.0, 8.0)	−1.6	(−2.3, −1.0)	<0.001	−8.0–4.7

**Bold** = not statistically significantly different at *p* < 0.05. Abbreviations: CI, confidence interval; diff, difference; LOA, limits of agreement; NSP, non-starch polysaccharides; WDR, weighed diet records. ^1^ Data are for the ‘fruit and vegetable adjusted’ EAT5 FFQ; ^2^ FFQ1 or FFQ2 was randomly chosen for each participant; ^3^ Geometric mean; ^4^ Paired *t*-test; ^5^ Bland-Altman limits of agreement [29].

**Table 2:** Nutrient correlations between the EAT5 FFQ and WDR (*n* = 100), and reproducibility correlations (*n* = 99) in 5-year old children ^1^.

Nutrient	Relative Validity ^2^			Reproducibility ^3^	
	EAT5 FFQ ^4^ vs. WDR	MSM Adjusted EAT5 FFQ ^5^ vs. WDR	Previous Studies ^6^	EAT5 FFQ1 vs. EAT5 FFQ2	Previous Studies ^7^
Energy (kJ)	0.32	0.32	0.19–0.66	0.88	0.29–0.73
Carbohydrate (g)	0.37	0.37	0.14–0.66	0.87	0.26–0.67
Fiber (g)	0.36	0.38	0.02–0.60	0.80	0.26–0.78
Total NSP (g)	0.38	0.39	NR	0.80	NR
Soluble NSP (g)	0.24	0.25	NR	0.80	NR
Insoluble NSP (g)	0.38	0.39	NR	0.80	NR

Abbreviations: NSP, non-starch polysaccharides; NR, not reported. ^1^ Data are for the ‘fruit and vegetable adjusted’ EAT5 FFQ; ^2^ Spearman’s correlation coefficients; ^3^ Intra-class correlation coefficients; ^4^ FFQ1 or FFQ2 was randomly chosen for each participant; ^5^ Using Multiple Source Method (MSM) [28] to adjust for the intra-individual variation occurring between the 3 days of diet records; ^6^ Inclusion of 12 studies with correlations for nutrients: three Spearman’s correlations [30,31,32], nine Pearson’s correlations [33,34,35,36,37,38,39,40,41]; ^7^ Inclusion of 5 studies with correlations for reproducibility for nutrients: one Intra-class correlation coefficients [31], four Pearson’s correlations [35,37,39,41].

**Table 3:** Nutrient cross-classification between EAT5 FFQ and WDR quartiles in 5-year old children (*n* = 100) ^1,2^.

Nutrient	Cross-Classification			
	% Correctly Classified ^3^	% Correct & Adjacent ^4^	% Grossly Misclassified ^5^	% Correct Extremes ^6^
*Chance*	*25%*	*62.5%*	*12.5%*	*12.5%*
Energy (kJ)	34	79	9	19
Carbohydrate (g)	36	76	6	23
Fiber (g)	28	76	4	18
Total NSP (g)	29	77	6	18
Soluble NSP (g)	32	69	5	19
Insoluble NSP (g)	28	76	4	18

Abbreviations: NSP, non-starch polysaccharides. ^1^ Data are for the ‘fruit and vegetable adjusted’ EAT5 FFQ; ^2^ FFQ1 or FFQ2 was randomly chosen for each participant; ^3^ % Correctly classified = percentage of children with WDR and FFQ intakes in the same quartile; ^4^ % Correct and adjacent = percentage of children with WDR and FFQ intakes in the same and adjacent quartiles; ^5^ % Grossly misclassified = percentage of children with WDR intakes in the highest quartile and FFQ intakes in the lowest quartile, or vice versa; ^6^ % Correctly classified to extreme quartiles = percentage of children with WDR and FFQ intakes correctly classified to the lowest and highest quartiles.

**Table 4:** Ability of the EAT5 FFQ to differentiate between quartiles of WDR intake, determined using actual values for surrogate categories (*n* = 100) ^1,2^.

Nutrient	*Quartiles Defined by*	Mean WDR Intake				*p* for Trend ^3^	*p* for Q1 vs. Q4 ^4^
		*Q1*	*Q2*	*Q3*	*Q4*		
Energy (kJ)	EAT5 FFQ	5414	5858	6321	6262	**0.004**	**0.001**
	WDR	4527	5505	6346	7477		
Carbohydrate (g)	EAT5 FFQ	164	186	193	199	**0.002**	**0.003**
	WDR	132	175	198	238		
Fiber (g)	EAT5 FFQ	16	18	19	21	**0.005**	**0.007**
	WDR	12	16	20	27		
Total NSP (g)	EAT5 FFQ	14	15	17	18	**0.001**	**0.002**
	WDR	10	14	17	23		
Soluble NSP (g)	EAT5 FFQ	5.7	6.6	6.6	7.2	**0.001**	**0.008**
	WDR	4.4	5.7	6.8	9.3		
Insoluble NSP (g)	EAT5 FFQ	8.1	9.1	9.5	11	**0.001**	**0.001**
	WDR	5.7	8.2	10	14		

**Bold** = statistically significant difference at *p* < 0.05. Abbreviations: NSP, non-starch polysaccharides. ^1^ Data are for the ‘fruit and vegetable adjusted’ EAT5 FFQ; ^2^ FFQ1 or FFQ2 was randomly chosen for each participant; ^3^ Significant difference in the trend across the quartiles (regression); ^4^ Significant differences between Q1 vs. Q4 (regression).

**Table 5:** Mean daily intake, mean difference and limits of agreement for food groups (energy contribution) of relevance to the gut microbiota according to WDR and EAT5 FFQ in 5-year old children (*n* = 100) ^1,2^.

Food Group	WDR		EAT5 FFQ		EAT5 FFQ vs. WDR			
	Mean ^3^	(95% CI)	Mean ^3^	(95% CI)	Mean Diff	(95% CI)	*p* ^4^	LOA ^5^
High fiber more healthy cereals (kJ)	858	(740, 996)	805	(689, 941)	−22	(−161, 118)	**0.758**	−1428–1385
Low fiber more healthy cereals (kJ)	498	(430, 577)	422	(360, 494)	−49	(−142, 44)	**0.298**	−986–888
High fiber less healthy cereals (kJ)	324	(268, 392)	290	(240, 350)	4.4	(−79, 88)	**0.917**	−838–847
Low fiber less healthy cereals (kJ)	315	(254, 391)	394	(336, 462)	188	(98, 279)	<0.001	−724–1101
Nuts and legumes (kJ)	133	(94, 188)	176	(138, 226)	68	(12, 123)	0.018	−493–628
Fruits (kJ)	474	(415, 541)	567	(499, 644)	113	(36, 190)	0.004	−662–889
Vegetables (kJ)	90	(73, 110)	86	(69, 106)	−4.4	(−32, 23)	**0.753**	−283–274
Potatoes (kJ)	142	(112, 181)	133	(111, 159)	11	(−28, 50)	**0.589**	−384–406
Dairy (kJ)	615	(544, 696)	765	(662, 884)	240	(125, 354)	<0.001	−912–1392
Yoghurt (kJ)	190	(161, 226)	193	(159, 233)	49	(17, 80)	0.003	−269–367
Meat, fish, egg (kJ)	468	(404, 542)	760	(672, 860)	320	(235, 405)	<0.001	−537–1176
Miscellaneous (kJ)	930	(828, 1044)	830	(734, 939)	−88	(−225, 48)	**0.201**	−1462–1285

**Bold** = not statistically significant at *p* < 0.05. Abbreviations: CI, confidence interval; diff, difference; LOA, limits of agreement. ^1^ Data are for the ‘fruit and vegetable adjusted’ EAT5 FFQ; ^2^ FFQ1 or FFQ2 was randomly chosen for each participant; ^3^ Geometric mean; ^4^ Paired *t*-test; ^5^ Bland-Altman limits of agreement [29].

**Table 6:** Food group (energy contribution) correlations between the EAT5 FFQ and WDR (*n* = 100), and reproducibility correlations (*n* = 99) in 5-year old children ^1^.

Food Group	Relative Validity ^2^	Reproducibility ^3^
	EAT5 FFQ ^4^ vs. WDR	EAT5 FFQ1 vs. EAT5 FFQ2
High fiber more healthy cereals (kJ)	0.37	0.84
Low fiber more healthy cereals (kJ)	0.35	0.91
High fiber less healthy cereals (kJ)	0.38	0.80
Low fiber less healthy cereals (kJ)	0.31	0.82
Nuts and legumes cereals (kJ)	0.45	0.69
Fruits (kJ)	0.42	0.83
Vegetables (kJ)	0.28	0.78
Potatoes (kJ)	0.51	0.57
Dairy (kJ)	0.50	0.89
Yoghurt (kJ)	0.54	0.81
Meat, fish, egg (kJ)	0.56	0.83
Miscellaneous (kJ)	0.28	0.84

^1^ Data are for the ‘fruit and vegetable adjusted’ EAT5 FFQ; ^2^ Spearman’s correlation coefficients; ^3^ Intra-class correlation coefficients; ^4^ FFQ1 or FFQ2 was randomly chosen for each participant.

**Table 7:** Food group (energy contribution) cross-classifications between EAT5 FFQ and WDR quartiles in 5-year old children (*n* = 100) ^1,2^.

Food Group	Cross-Classification			
	% Correctly Classified ^3^	% Correct & Adjacent ^4^	% Grossly Misclassified ^5^	% Correct Extremes ^6^
*Chance*	*25%*	*62.5%*	*12.5%*	*12.5%*
High fiber more healthy cereals (kJ)	36	75	5	21
Low fiber more healthy cereals (kJ)	37	72	5	20
High fiber less healthy cereals (kJ)	33	78	7	22
Low fiber less healthy cereals (kJ)	30	69	6	20
Nuts and legumes (kJ)	38	78	4	25
Fruits (kJ)	39	74	5	23
Vegetables (kJ)	28	69	5	17
Potatoes (kJ)	41	82	3	24
Dairy (kJ)	42	84	6	24
Yoghurt (kJ)	47	83	4	29
Meat, fish, egg (kJ)	51	79	2	29
Miscellaneous (kJ)	32	71	9	20

^1^ Data are for the ‘fruit and vegetable adjusted’ EAT5 FFQ; ^2^ FFQ1 or FFQ2 was randomly chosen for each participant; ^3^ % Correctly classified = percentage of children with WDR and FFQ intakes in the same quartile; ^4^ % Correct and adjacent = percentage of children with WDR and FFQ intakes in the same and adjacent quartiles; ^5^ % Grossly misclassified = percentage of children with WDR intakes in the highest quartile and FFQ intakes in the lowest quartile and vice versa; ^6^ % Correctly classified to extreme quartiles = percentage of children with WDR and FFQ intakes correctly classified to the lowest and highest quartiles.

**Table 8:** Ability of the EAT5 FFQ to differentiate between quartiles of WDR food group intake (energy contribution), determined using actual values for surrogate categories (*n* = 100) ^1,2^.

Food Group	*Quartiles Defined by*	Mean WDR Intake				*p* for Trend ^3^	*p* for Q1 vs. Q4 ^4^
		*Q1*	*Q2*	*Q3*	*Q4*		
High fiber more healthy cereals (kJ)	EAT5 FFQ	719	1051	1070	1347	**<0.001**	**<0.001**
	WDR	357	780	1161	1890		
Low fiber more healthy cereals (kJ)	EAT5 FFQ	431	556	668	781	**0.002**	**0.004**
	WDR	172	422	642	1199		
High fiber less healthy cereals (kJ)	EAT5 FFQ	235	329	496	514	**0.002**	**0.009**
	WDR	30	183	411	934		
Low fiber less healthy cereals (kJ)	EAT5 FFQ	152	334	393	386	**0.028**	**0.033**
	WDR	0	120	314	856		
Nuts and legumes (kJ)	EAT5 FFQ	76	120	236	424	**<0.001**	**<0.001**
	WDR	0	55	199	601		
Fruits (kJ)	EAT5 FFQ	388	576	550	712	**0.001**	**<0.001**
	WDR	224	439	615	949		
Vegetables (kJ)	EAT5 FFQ	72	141	152	156	**0.016**	**0.015**
	WDR	28	70	124	299		
Potatoes (kJ)	EAT5 FFQ	68	136	174	294	**<0.001**	**<0.001**
	WDR	1	68	165	439		
Dairy (kJ)	EAT5 FFQ	478	606	835	910	**<0.001**	**<0.001**
	WDR	257	519	843	1208		
Yoghurt (kJ)	EAT5 FFQ	65	138	220	291	**<0.001**	**<0.001**
	WDR	0	92	215	394		
Meat, fish, egg (kJ)	EAT5 FFQ	287	574	598	829	**<0.001**	**<0.001**
	WDR	186	388	621	1094		
Miscellaneous (kJ)	EAT5 FFQ	849	1021	1213	1213	**0.010**	**0.021**
	WDR	484	854	1142	1816		

**Bold** = Statistically significant difference at *p* < 0.05. ^1^ Data are for the ‘fruit and vegetable adjusted’ EAT5 FFQ; ^2^ FFQ1 or FFQ2 was randomly chosen for each participant; ^3^ Significant difference in the trend across the quartiles (regression); ^4^ Significant differences between Q1 vs. Q4 (regression).

## Supplementary Materials

The following are available online at http://www.mdpi.com/2072-6643/10/11/1627/s1, Table S1: Mean daily intake, mean difference and limits of agreement for selected nutrients of relevance to the gut microbiota according to WDR and crude EAT5 FFQ in 5-year old children (*n* = 100), Table S2. Mean daily intake, mean difference and limits of agreement for other nutrients according to WDR, crude EAT5 FFQ and ‘fruit and vegetable adjusted’ EAT5 FFQ in 5-year old children (*n* = 100), Table S3. Nutrients of relevance to the gut microbiota correlations between the crude EAT5 FFQ 1 and WDR (*n* = 100), and reproducibility (*n* = 99) in 5-year old children, Table S4. Other nutrient correlations between the crude EAT5 FFQ and WDR (*n* = 100), and reproducibility (*n* = 99) in 5-year old children, Table S5. Nutrients of relevance to the gut microbiota–cross-classification between the crude EAT5 FFQ and WDR in 5 year old children (*n* = 100), Table S6: Other nutrients cross-classification between the crude EAT5 FFQ and WDR in 5-year old children (*n* = 100). Table S7: Mean daily intake, mean difference and limits of agreement for food groups (in grams) of relevance to the gut microbiota according to WDR and EAT5 FFQ in 5-year old children (*n* = 100). Table S8: Food group (in grams) correlations between the EAT5 FFQ and WDR (*n* = 100), and reproducibility correlations (*n* = 99) in 5-year old children. Table S9: Food group (in grams) cross-classifications between EAT5 FFQ and WDR quartiles in 5-year old children (*n* = 100). Figure S1: Bland-Altman plots of food group intakes (energy contribution) from a randomly chosen FFQ1 or FFQ2 EAT5 FFQ (‘fruit and vegetable adjusted’) and WDR: (a) High fiber more healthy cereals; (b) Low fiber more healthy cereals; (c) High fiber less healthy cereals; (d) Low fiber less healthy cereals; (e) Fruit; and (f) Vegetables.
