# Supplementary material for: Relative Validity and Reproducibility of a Food Frequency Questionnaire to Assess Nutrients and Food Groups of Relevance to the Gut Microbiota in Young Children
Source: Nutrients. 2018 Nov 2;10(11):1627. doi: 10.3390/nu10111627 (PMC6266661; doi:10.3390/nu10111627)
Supplement: Supplementary file 1 [file nutrients-10-01627-s001.pdf]

nutrients

MDPI

Supplementary material

Table S1. Mean daily intake, mean difference and limits of agreement for selected nutrients of relevance to the gut microbiota according to WDR and crude EAT5 FFQ in 5-year old children (n = 100). [1]

	WDR		Crude FFQ		Crude FFQ vs WDR			
**Nutrient**	**Mean [2]**	**(95% CI)**	**Mean [2]**	**(95% CI)**	**Mean diff**	**(95% CI)**	***p* [3]**	**LOA [4]**
Energy (kJ)	5845	(5613, 6086)	6866	(6488, 7267)	1187	(774, 1595)	<0.001	-2952-5322
Carbohydrate (g)	181	(173, 190)	201	(191, 213)	24	(12, 36)	<0.001	-98-146
Fiber (g)	18	(17, 19)	23	(22, 25)	5.5	(4.0, 7.1)	<0.001	-10-21
Total NSP (g)	15	(14, 16)	19	(18, 20)	4.1	(2.8, 5.3)	<0.001	-8.6-16.7
Soluble NSP (g)	6.3	(5.9, 6.6)	9.9	(9.2, 10.7)	4.0	(3.3, 4.7)	<0.001	-3.4-11.4
Insoluble NSP (g)	8.9	(8.3, 9.6)	9.1	(8.5, 9.7)	0.02	(-0.6, 0.7)	**0.945**	-6.7-6.7

Bold = not statistically significant at *p*<0.05. Abbreviations: CI, confidence interval; diff, difference; LOA, limits of agreement; NSP, non-starch polysaccharides. [1] FFQ1 or FFQ2 was randomly chosen for each participant; [2] Geometric mean; [3] Paired t-test; [4] Bland-Altman limits of agreement.

Table S2. Mean daily intake, mean difference and limits of agreement for other nutrients according to WDR, crude EAT5 FFQ and FV-EAT5 FFQ in 5-year old children (n = 100). [1]

	WDR		Crude FFQ		FV-FFQ		FV-FFQ vs WDR			
**Nutrient**	**Mean [2]**	**(95% CI)**	**Mean [2]**	**(95% CI)**	**Mean [2]**	**(95% CI)**	**Mean diff**	**(95% CI)**	***p* [3]**	**LOA [4]**
Protein (g)	51	(49, 53)	65	(61, 70)	63	(59, 67)	14	(9.6, 18)	<0.001	-28-55
Total fat (g)	50	(47, 53)	62	(58, 66)	59	(56, 64)	11	(6.7, 16)	<0.001	-35-58
Sugar (g)	77	(72, 82)	94	(87, 100)	82	(76, 89)	6.8	(0.4, 13.3)	0.039	-58-72
Sodium (mg)	1560	(1453, 1675)	1901	(1790, 2020)	1793	(1686, 1908)	233	(95, 371)	0.001	-1155-1621
Calcium (mg)	593	(548, 640)	730	(669, 795)	686	(628, 751)	123	(58, 187)	<0.001	-532-777
Iron (mg)	8.8	(8.2, 9.5)	10.6	(9.9, 11.3)	9.8	(9.1, 10.5)	0.9	(0.1, 1.6)	0.021	-6.4-8.2
Vitamin C (mg)	63	(56, 70)	107	(95, 119)	74	(65, 84)	16	(6.1, 25)	0.002	-81-113

Bold = not statistically significant at *p*<0.05. Abbreviations: FV, fruit and vegetables adjusted; CI, confidence interval; diff, difference; LOA, limits of agreement. [1] FFQ1 or FFQ2 was randomly chosen for each participant; [2] Geometric mean; [3] Paired t-test; [4] Bland-Altman limits of agreement.

Table S3. Nutrients of relevance to the gut microbiota correlations between the crude EAT5 FFQ and WDR (n = 100), and reproducibility (n = 99) in 5-year old children.

	Relative validity [1]	Reproducibility [2]
**Nutrient**	**Crude FFQ vs WDR [3]**	**Crude FFQ1 vs Crude FFQ2**
Energy (kJ)	0.32	0.88
Carbohydrate (g)	0.36	0.86
Fiber (g)	0.33	0.82
Total NSP (g)	0.32	0.82
Soluble NSP (g)	0.18	0.84
Insoluble NSP (g)	0.34	0.80

Abbreviations: NSP, non-starch polysaccharides. [1] Spearman's correlation coefficients; [2] Intra-class correlation coefficients; [3] FFQ1 or FFQ2 was randomly chosen for each participant.

Table S4. Other nutrients correlations between the crude EAT5 FFQ and WDR (n = 100), and reproducibility (n = 99) in 5-year old children.

	Relative validity [1]		Reproducibility [2]
**Nutrient**	**Crude FFQ vs WDR [3]**	**FV-FFQ vs WDR [3]**	**FV-FFQ1 vs FV-FFQ2**
Protein (g)	0.43	0.41	0.87
Total fat (g)	0.25	0.26	0.85
Sugar (g)	0.36	0.42	0.88
Sodium (mg)	0.24	0.23	0.87
Calcium (mg)	0.49	0.50	0.90
Iron (mg)	0.46	0.45	0.89
Vitamin C (mg)	0.51	0.49	0.81

Abbreviations: FV, fruit and vegetables adjusted. [1] Spearman's correlation coefficients; [2] Intra-class correlation coefficients; [3] FFQ1 or FFQ2 was randomly chosen for each participant.

Table S5. Nutrients of relevance to the gut microbiota cross-classifications between the crude EAT5 FFQ and WDR in 5-year old children (n = 100). [1]

	Cross-classification (Crude FFQ vs WDR)			
**Nutrient**	**% Correctly classified [2]**	**% Correct & adjacent [3]**	**% Grossly misclassified [4]**	**% Correct extremes [5]**
Chance	*25%*	*62.5%*	*12.5%*	*12.5%*
Energy (kJ)	32	79	9	18
Carbohydrate (g)	38	77	5	25
Fiber (g)	34	79	5	20
Total NSP (g)	28	75	9	16
Soluble NSP (g)	28	69	9	19
Insoluble NSP (g)	38	75	7	20

Abbreviations: NSP, non-starch polysaccharides. [1] FFQ1 or FFQ2 was randomly chosen for each participant; [2] % Correctly classified = percentage of children with WDRs and FFQ intakes in the same quartile; [3] % Correct and adjacent = percentage of children with WDRs and FFQ intakes in the same and 1 adjacent quartile; [4] % Grossly misclassified = percentage of children with WDRs intakes in the highest quartile and FFQ intakes in the lowest quartile and vice versa; [5] % Correctly classified to extreme quartiles = percentage of children with WDRs and FFQ intakes correctly classified to the lowest and highest quartiles.

Table S6. Other nutrients cross-classifications between the crude EAT5 FFQ and WDR in 5-year old children (n = 100). [1]

Nutrient	Cross-classification (FV-FFQ vs WDR)			
	**% Correctly classified** [2]	**% Correct & adjacent** [3]	**% Grossly misclassified** [4]	**% Correct extremes** [5]
Chance	*25%*	*62.5%*	*12.5%*	*12.5%*
Protein (g)	42	78	8	24
Total fat (g)	29	67	6	20
Sugar (g)	40	78	4	24
Sodium (mg)	32	71	9	17
Calcium (mg)	43	84	3	27
Iron (mg)	39	78	5	24
Vitamin C (mg)	31	78	1	22

Abbreviations: FV, fruit and vegetables adjusted. [1] FFQ1 or FFQ2 was randomly chosen for each participant; [2] % Correctly classified = percentage of children with WDRs and FFQ intakes in the same quartile; [3] % Correct and adjacent = percentage of children with WDRs and FFQ intakes in the same and 1 adjacent quartile; [4] % Grossly misclassified = percentage of children with WDRs intakes in the highest quartile and FFQ intakes in the lowest quartile and vice versa; [5] % Correctly classified to extreme quartiles = percentage of children with WDRs and FFQ intakes correctly classified to the lowest and highest quartiles.

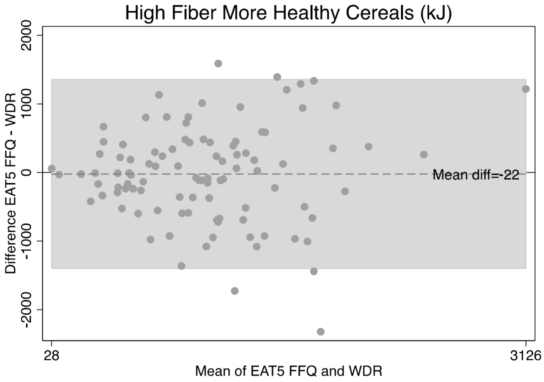

(**a**)

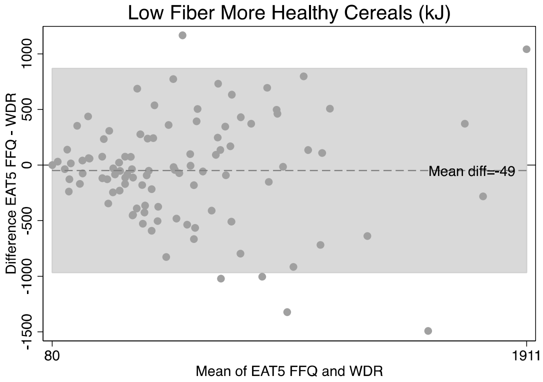

(**b**)

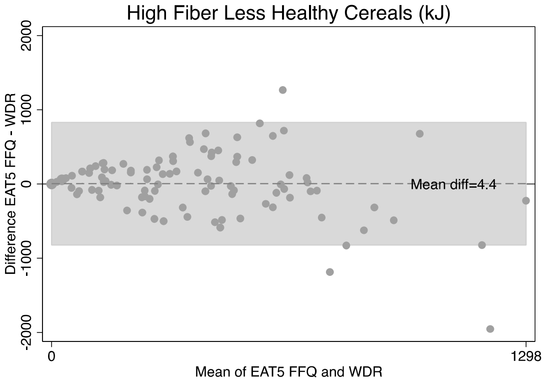

(**c**)

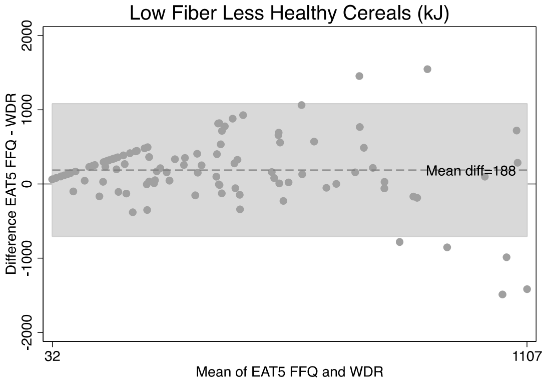

(**d**)

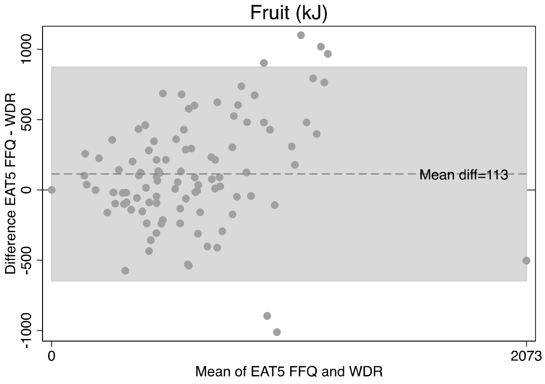

(**e**)

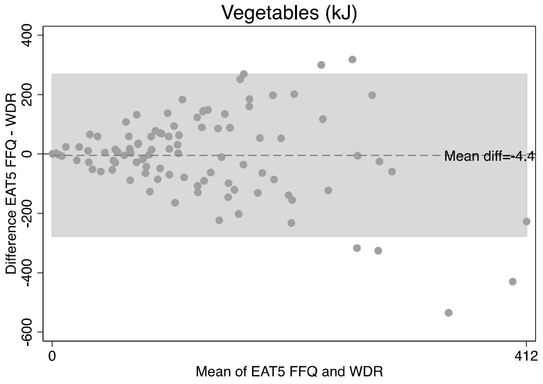

(**f**)

Figure S1. Bland-Altman plots of food group intakes (energy contribution) from a randomly chosen FFQ1 or FFQ2 EAT5 FFQ ('fruit and vegetable adjusted') and WDR: (a) High fiber more healthy cereals; (b) Low fiber more healthy cereals; (c) High fiber less healthy cereals; (d) Low fiber less healthy cereals; (e) Fruit; and (f) Vegetables.

Table S7. Mean daily intake, mean difference and limits of agreement for food groups (in grams) of relevance to the gut microbiota according to WDR and EAT5 FFQ in 5-year old children (n = 100). [1, 2]

Food group acronym	WDR		EAT5 FFQ		EAT5 FFQ vs WDR			
	Mean [3]	(95% CI)	Mean [3]	(95% CI)	Mean diff	(95% CI)	*p* [4]	LOA [5]
High fiber more healthy cereals (g)	75	(65, 88)	72	(62, 85)	1.7	(-11, 14)	**0.794**	-124, 128
Low fiber more healthy cereals (g)	51	(43, 60)	81[6]	(70, 94)	39	(25, 53)	<0.001	-105, 183
High fiber less healthy cereals (g)	19	(16, 23)	16	(13, 20)	0.3	(-5.4, 5.9)	**0.922**	-57, 57
Low fiber less healthy cereals (g)	19	(15, 24)	28	(23, 33)	17	(10, 24)	<0.001	-52, 86
Nuts and legumes (g)	10	(7, 14)	16	(12, 21)	11	(5.3, 16)	<0.001	-43, 64
Fruits (g)	178	(155, 204)	214	(188, 243)	47	(18, 76)	0.002	-246, 339
Vegetables (g)	70	(59, 84)	55	(43, 68)	-5.9	(-21, 9.0)	**0.433**	-156, 144
Potatoes (g)	34	(28, 42)	19	(16, 22)	-9.1	(-15, -3.4)	0.002	-67, 49
Dairy (g)	139	(118, 163)	169	(138, 207)	70	(37, 103)	<0.001	-262, 403
Yoghurt (g)	61	(51, 71)	55	(45, 66)	8.6	(-0.7, 18)	**0.068**	-85, 102
Meat, fish, egg (g)	68	(60, 78)	89	(78, 100)	24	(15, 32)	<0.001	-64, 111
Miscellaneous (g)	85	(71, 100)	100	(86, 117)	15	(-2.7, 33)	**0.094**	-167, 197

Bold = not statistically significant at $p<0.05$. Abbreviations: CI, confidence interval; diff, difference; LOA, limits of agreement. [1] Data are for the 'fruit and vegetable adjusted' EAT5 FFQ; [2] FFQ1 or FFQ2 was randomly chosen for each participant; [3] Geometric mean; [4] Paired t-test; [5] Bland-Altman limits of agreement; [6] Contains porridge in this food group which includes water in the FFQ nutrient line calculation but in the food diary, porridge may be dried oats/ rolled oats on its own.

Table S8. Food group (in grams) correlations between the EAT5 FFQ and WDR (n = 100), and reproducibility correlations (n = 99) in 5-year old children. [1]

	Relative validity [2]	Reproducibility [3]
**Food group**	**EAT5 FFQ [4] vs WDR**	**EAT5 FFQ1 vs EAT5 FFQ2**
High fiber more healthy cereals (g)	0.40	0.83
Low fiber more healthy cereals (g)	0.39	0.88
High fiber less healthy cereals (g)	0.37	0.79
Low fiber less healthy cereals (g)	0.28	0.81
Nuts and legumes (g)	0.38	0.70
Fruits (g)	0.33	0.80
Vegetables (g)	0.29	0.86
Potatoes (g)	0.48	0.63
Dairy (g)	0.62	0.93
Yoghurt (g)	0.54	0.81
Meat, fish, egg (g)	0.63	0.82
Miscellaneous (g)	0.53	0.84

[1] Data are for the 'fruit and vegetable adjusted' EAT5 FFQ; [2] Spearman's correlation coefficients; [3] Intra-class correlation coefficients; [4] FFQ1 or FFQ2 was randomly chosen for each participant.

Table S9. Food group (in grams) cross-classifications between EAT5 FFQ and WDR quartiles in 5-year old children (n = 100). [1, 2]

	Cross-classification			
**Food group**	**% Correctly classified [3]**	**% Correct & adjacent [4]**	**% Grossly misclassified [5]**	**% Correct extremes [6]**
Chance	*25%*	*62.5%*	*12.5%*	*12.5%*
High fiber more healthy cereals (g)	35	74	7	20
Low fiber more healthy cereals (g)	37	79	6	23
High fiber less healthy cereals (g)	37	75	5	22
Low fiber less healthy cereals (g)	33	70	6	21
Nuts and legumes (g)	38	78	8	22
Fruits (g)	40	71	7	20
Vegetables (g)	30	70	8	18
Potatoes (g)	40	83	3	25
Dairy (g)	53	86	3	31
Yoghurt (g)	51	83	4	30
Meat, fish, egg (g)	46	84	2	30
Miscellaneous (g)	41	85	4	25

[1] Data are for the 'fruit and vegetable adjusted' EAT5 FFQ; [2] FFQ1 or FFQ2 was randomly chosen for each participant; [3] % Correctly classified = percentage of children with WDR and FFQ intakes in the same quartile; [4] % Correct and adjacent = percentage of children with WDR and FFQ intakes in the same and adjacent quartiles; [5] % Grossly misclassified = percentage of children with WDR intakes in the highest quartile and FFQ intakes in the lowest quartile and vice versa; [6] % Correctly classified to extreme quartiles = percentage of children with WDR and FFQ intakes correctly classified to the lowest and highest quartiles.
